# Phenotype is sustained during hospital readmissions following treatment for complicated severe malnutrition among Kenyan children: A retrospective cohort study

**DOI:** 10.1111/mcn.12913

**Published:** 2019-11-22

**Authors:** Gerard Bryan Gonzales, Moses M. Ngari, James M. Njunge, Johnstone Thitiri, Laura Mwalekwa, Neema Mturi, Martha K. Mwangome, Caroline Ogwang, Amek Nyaguara, James A. Berkley

**Affiliations:** ^1^ Laboratory of Gastroenterology, Department of Internal Medicine and Paediatrics, Faculty of Medicine and Health Sciences Ghent University Ghent Belgium; ^2^ VIB Inflammation Research Centre Ghent Belgium; ^3^ Department of Translational Medicine Hospital for Sick Children Toronto Canada; ^4^ The Childhood Acute Illness & Nutrition (CHAIN) Network Nairobi Kenya; ^5^ KEMRI‐Wellcome Trust Research Programme Kilifi Kenya; ^6^ Centre for Tropical Medicine & Global Health, Nuffield Department of Medicine University of Oxford Oxford UK

**Keywords:** complicated SAM, kwashiorkor, malnutrition, marasmus, relapse, readmission, severe acute malnutrition

## Abstract

Hospital readmission is common among children with complicated severe acute malnutrition (cSAM) but not well‐characterised. Two distinct cSAM phenotypes, marasmus and kwashiorkor, exist, but their pathophysiology and whether the same phenotype persists at relapse are unclear. We aimed to test the association between cSAM phenotype at index admission and readmission following recovery. We performed secondary data analysis from a multicentre randomised trial in Kenya with 1‐year active follow‐up. The main outcome was cSAM phenotype upon hospital readmission. Among 1,704 HIV‐negative children with cSAM discharged in the trial, 177 children contributed a total of 246 readmissions with cSAM. cSAM readmission was associated with age<12 months (*p* = .005), but not site, sex, season, nor cSAM phenotype. Of these, 42 children contributed 44 readmissions with cSAM that occurred after a monthly visit when SAM was confirmed absent (cSAM relapse). cSAM phenotype was sustained during cSAM relapse. The adjusted odds ratio for presenting with kwashiorkor during readmission after kwashiorkor at index admission was 39.3 [95% confidence interval (95% CI) [2.69, 1,326]; *p* = .01); and for presenting with marasmus during readmission after kwashiorkor at index admission was 0.02 (95% CI [0.001, 0.037]; *p* = .01). To validate this finding, we examined readmissions to Kilifi County Hospital, Kenya occurring at least 2 months after an admission with cSAM. Among 2,412 children with cSAM discharged alive, there were 206 readmissions with cSAM. Their phenotype at readmission was significantly influenced by their phenotype at index admission (*p* < .001). This is the first report describing the phenotype and rate of cSAM recurrence.

**Key messages**

- Severe malnutrition phenotype was persistent such that a child readmitted for kwashiorkor would be likely to be readmitted with kwashiorkor again.
- Readmission with complicated severe malnutrition after clinical stabilisation occurred in 10.2% of children at a rate of 15.9 (95% CI [13.9, 18.0]) per 100 child years.
- Severe malnutrition phenotype did not affect the readmission rate, but children below 12 months old had higher risks of readmission with complicated severe acute malnutrition.

## 1 INTRODUCTION

Severe acute malnutrition (SAM) remains a serious public health burden and contributes to more than a third of all deaths among children under 5 years (Black et al., 2013; UNICEF/WHO/World Bank, 2017). In 2016, global estimates suggested that nearly 52 million children under 5 years old were wasted (acutely malnourished), of which 17 million were severely wasted (UNICEF/WHO/World Bank, 2017). SAM increases susceptibility to diseases and infections. Hence, many of these children also suffer from common illnesses, which can impede their recovery. The combination of SAM and infection, known as complicated SAM (cSAM), if untreated, forms a vicious downward spiral where both conditions exacerbate each other (Ambrus & Ambrus, 2004). SAM is clinically categorised into two entirely different phenotypes: marasmus (severe wasting), which is characterised by muscle atrophy; and kwashiorkor (oedematous malnutrition), characterized by oedema, fatty liver, hair depigmentation, desquamating skin lesions, and behavioural changes; some children present with both phenotypes concurrently (“marasmic–kwashiorkor”; Bhutta et al., 2017). In the absence of understanding of their pathophysiological differences, treatment protocols for cSAM are the same for both phenotypes (Manary & Brewster, 2000).

Current guidelines recommend that children admitted with cSAM are discharged and transferred to community care when their medical complications, including oedema, are resolving, they are clinically well and alert, and have good appetite, rather than hospital discharge being based on anthropometric criteria (WHO, 2019). However, children with cSAM experience a high rate of mortality and readmission in the months following hospital discharge especially in sub‐Saharan Africa (Berkley et al., 2016; Chhibber et al., 2015; Dubray et al., 2008; Kerac et al., 2009; Moisi et al., 2011; Ngari et al., 2017; Trehan et al., 2013; Veirum, Sodeman, Biai, Hedegård, & Aaby, 2007; Wiens et al., 2013; Wiens et al., 2015). Recent systematic reviews reported paediatric post‐discharge mortality rates in resource‐poor countries of up to 18% that may exceed in‐hospital mortality rates in many settings (Nemetchek et al., 2018; Wiens et al., 2013). Further, post‐discharge relapse is poorly defined and infrequently systematically measured in programmes, and therefore less understood. A recent systematic review showed that the proportion of children who relapse following SAM treatment varies greatly from 0 to 37% across different lengths of time following discharge and with varying loss to follow‐up, but with the highest proportions occurring in the first 6 months postdischarge (Stobaugh et al., 2018). Such relapse rates are higher when the group with moderate acute malnutrition are considered. The review also highlighted the lack of a standard definition of relapse that limits comparability even among the few studies that have quantified post‐discharge relapse (Stobaugh et al., 2018). Low anthropometry at admission and discharge following an acute illness were highlighted as risk markers for relapse.

Most research on SAM has focussed on its causes, treatment, and short‐term consequences, whereas assessment of overall health and nutrition after discharge either from a hospital or community‐based (Community‐Based Management of Acute Malnutrition) nutrition programmes is limited. Furthermore, only few studies that evaluated post‐discharge outcomes have focussed on relapse to cSAM, which can be defined as a child presenting with cSAM following discharge (or default) from cSAM treatment (Stobaugh et al., 2018). Data on hospital readmissions with SAM following both clinical and nutritional rehabilitation is less common. It is also unclear whether a child presenting with one phenotype of SAM will present the same phenotype upon relapse to SAM. This knowledge could help better understand the natural history of the two phenotypes.

We evaluated readmissions with cSAM among children who were discharged after index hospitalisation with cSAM using data from a multicentre randomised controlled trial with active follow‐up for 1 year after discharge (Berkley et al., 2016) to determine whether there was an association between a child's phenotype at index admission and the phenotype at readmission. Findings on association between the index admission and readmission phenotypes were validated using data from a longer term clinical and demographic surveillance at one site in Kenya (Scott et al., 2012). We also estimated the incidence of readmission with cSAM in the randomised trial.

## 2 METHODS

### 2.1 Study design and settings

We performed secondary analysis of data from a randomised controlled trial that tested the efficacy of daily co‐trimoxazole prophylaxis in reducing post‐discharge mortality among HIV‐uninfected children with cSAM in two urban (Mombasa and Nairobi) and two rural (Kilifi and Malindi) hospitals in Kenya (Berkley et al., 2016). Discharge was standardised across site, based on World Health Organization (WHO) guidelines (2013) through training and monitoring. Children with cSAM were actively linked to therapeutic and supplementary feeding programmes upon hospital discharge. Children were then actively followed up for a total of 1 year, monthly in the first 6 months, and every 2 months until Month 12 for growth, readmission, or death. Study participants were traced at home if they defaulted and the loss to follow‐up was minimal (5%). The trial intervention had no overall effect on reducing mortality or hospital readmission.

For validation, we analysed a data set of children admitted to Kilifi County Hospital (KCH), Kenya from 2002 to 2016 with cSAM, and subsequent readmissions at KCH were detected through linkage with a unique identifier on a population database for those living within the Kilifi Health and Demographic Surveillance System (KHDSS) area, with quarterly household enumeration, as previously described (Ngari et al., 2018b; Scott et al., 2012). All the study hospitals offered inpatient and outpatient care according to Kenya (2009) national and WHO (2013) guidelines, and discharge was standardised based on WHO guidelines (2013). Children with cSAM were actively linked to therapeutic and supplementary feeding programmes upon hospital discharge. Missing follow‐up visits and needing to be traced by a fieldworker making a home visit was associated with readmission (with any condition, not just complicated SAM) and death, as previously published (Berkley et al., 2016; Ngari et al., 2018a).

### 2.2 Study population

The randomised clinical trial recruited children with cSAM aged 2–59 months who had completed the initial “stabilisation” phase of cSAM inpatient treatment. Children were eligible for the trial if mid upper arm circumference (MUAC) <11.5 cm for those aged 6–59 months, MUAC <11 cm for those aged <6 months, or having nutritional oedema at any age (defined in 2013 WHO guidelines). HIV‐infected children were excluded because they would already be on open‐label co‐trimoxazole prophylaxis. Children were included in the analysis if they were readmitted with cSAM. Children with SAM but not needing clinical care were therefore not included in the analysis.

From the KCH admissions database, we included children admitted with cSAM between 2002 and 2016 aged 2–59 months and discharged alive. The cSAM definition used among the KCH study participants was the same as for the trial participants. Acquiring an illness postdischarge is common among children with SAM but it is also possible that a subclinical condition persisted even after clinical care, which either only manifested or worsened postdischarge. As such, this would not be a new episode of disease. To reduce this bias for the analysis of readmission phenotype for the KCH admissions database, which did not have active follow‐up of patients post‐discharge, we limited the analysis to readmissions that occurred at least 2 months after discharge from the hospital. Children who did not require hospitalisation or those that were hospitalised in other hospital were not included in the analysis.

### 2.3 Variables

The primary outcome was hospital readmission SAM phenotype (kwashiorkor or marasmus). Kwashiorkor was regarded as having nutritional oedema regardless of wasting (i.e., marasmic–kwashiorkor). Children without nutritional oedema and with either MUAC <11.5 cm (or<11 cm if age <6 months) or weight‐for‐length/height <−3 were considered as marasmus. The other outcome of interest was incidence of readmission with cSAM in the randomised trial. The exposures examined were age, sex, SAM phenotype at index admission, recruitment site (for the clinical trial), season (rainy or dry) at admission, and randomisation arm (for the clinical trial). To account for seasonality of admissions, rainy season was considered as admission during March to May and October to December, whereas dry season was the remaining months of the year (Yang, Seager, Cane, & Lyon, 2014).

### 2.4 Data sources and measurement

In both the trial and at KCH, children were weighed using electronic scales (seca 825), length (children <2 years) was measured using an infantometer (seca 416), height (children 2 or more years old) using a stadiometer (seca 215), and MUAC using a nonstretch dedicated insertion tape Teaching‐Aid at Low Cost (TALC). Weight‐for‐length/height and length‐for‐age z‐scores were calculated using the 2006 WHO children growth references. Kwashiorkor was diagnosed by trained clinicians as pitting oedema: generalised on both feet, legs, hands, arms, and face; both feet plus lower legs, hands, or lower arms; or both feet only. Children recruited in the randomised trial were in follow‐up for 12 months with anthropometric measurements taken at every scheduled visit and during hospital readmission. For children admitted to KCH, anthropometric measurements were conducted at each admission, and there were no planned research follow‐ups with anthropometry.

### 2.5 Study size

**Figure 1:**
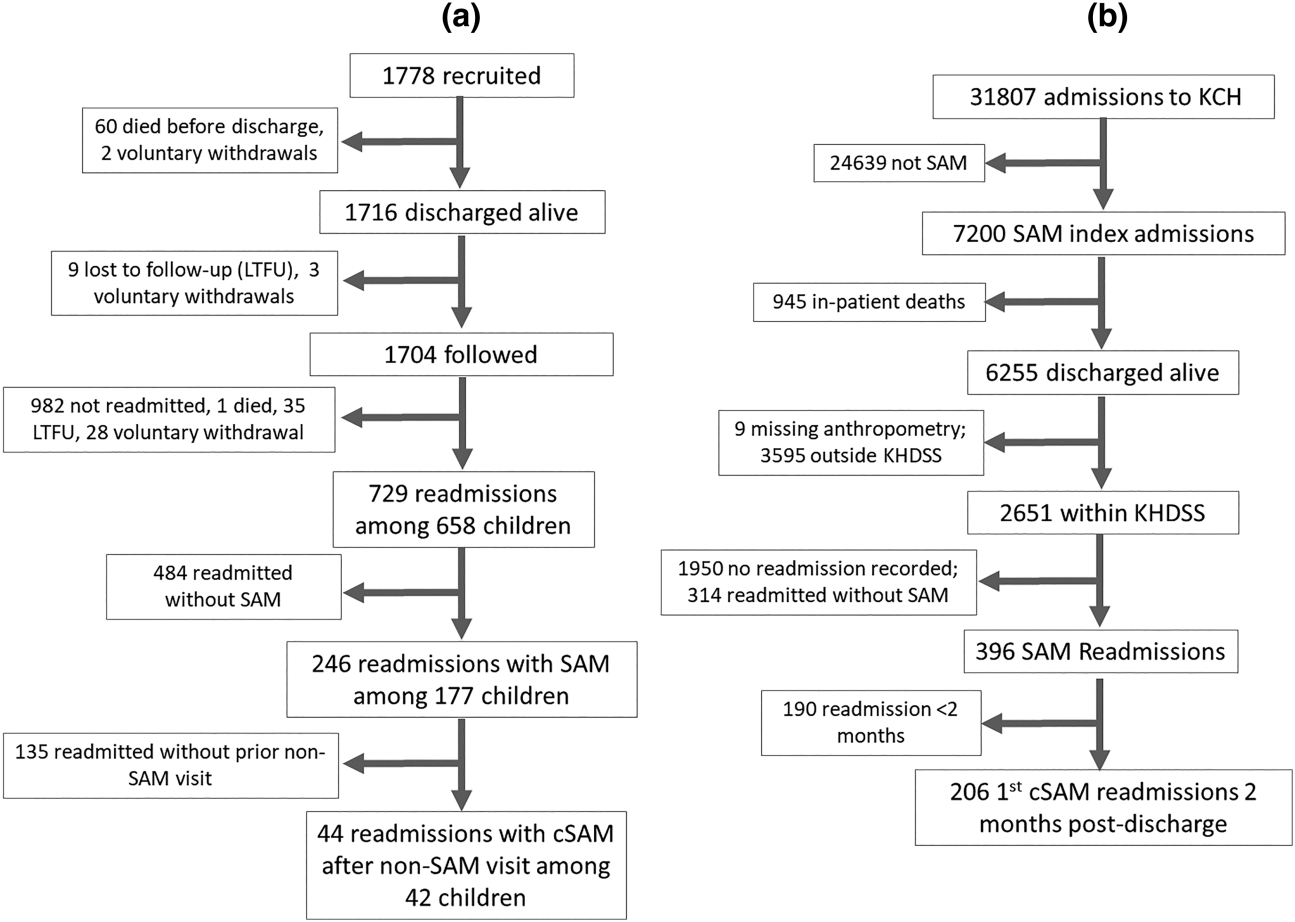
Recruitment flow diagram for the secondary analysis of the clinical trial (a) and the admissions data obtained from the Kilifi County Hospital (b). cSAM, complicated severe acute malnutrition; KCH, Kilifi County Hospital; KHDSS, Kilifi Health and Demographic Surveillance System; SAM, severe acute malnutrition

All children recruited in the trial and admitted at KCH with cSAM during the study period were included in the analysis. Therefore, no formal sample size was calculated. For the randomised trial, the sample size used had 90% power to detect a one‐third reduction in mortality, accounting for loss to follow‐up, assuming 15% mortality in the control arm. For the analysis of admission data from the KCH, all SAM admissions were included in the analysis. A total of 1,778 participants were enrolled in the randomised trial, of whom 1,704 were discharged alive and followed up (Figure 1a). In the KCH validation data set, 2,615 children were admitted with cSAM who were resident within the KHDSS (Figure 1b).

### 2.6 Data analysis

Data analyses were performed using R statistical software version 3.6 (R: A language and environment for statistical computing, 2009). Extreme weight‐for‐length/height *z*‐score ±6 from the median *z*‐score of 0 were excluded unless the child had kwashiorkor. For the children admitted at KCH, any admission with missing anthropometric measurements were excluded.

In order to assess the association between cSAM phenotype at index admission and the phenotype during cSAM readmission, we identified children from the randomised trial who were readmitted with cSAM after they had been non‐SAM in any of the monthly visits prior to their readmission with cSAM.

Because of this condition, the time elapsed from hospital discharge to readmission was also influenced by the rate of recovery of the child from SAM. Hence, instead of a time‐to‐event analysis, a mixed logistic regression model was used to evaluate the association between cSAM phenotype at index admission and readmission with site of recruitment set as random effect. The adjusted odds ratios for readmission with kwashiorkor (versus no readmission with kwashiorkor) and readmission with marasmus (versus no readmission with marasmus) were calculated separately.

To validate findings regarding the effect of cSAM phenotype at index admission on phenotype at subsequent admissions from the randomised trial, we examined data obtained from all cSAM admissions at the KCH. We used logistic regression to validate whether there was an association between index readmission and readmission cSAM phenotypes.

Using the clinical trial data only, the incidence of readmission with cSAM in the randomised trial was calculated as the number of readmissions with cSAM per 100 child‐years of observation: the time from date of index discharge to date of death, loss to follow up, or completing study. Since post‐discharge deaths and loss to follow‐up would preclude the probability of observing hospital readmission, we treated these events as competing risks. Then, to examine the association between SAM phenotype at index admission on the hospital readmission rate, we used the Fine‐Gray competing risk analysis method (Fine & Gray, 1999) implemented using the *timereg* package for R (Scheike & Zhang, 2011) instead of the more conservative Cox regression models and reported the effects using subdistribution hazards ratios. Because a child could have more than one hospital readmission, the model used accounted for multiple events and was adjusted for age, sex, randomised arm, and site with robust standard errors.

### 2.7 Study approvals

The trial and secondary analyses were approved by the Kenyan national ethics committee, KEMRI‐ SERU (SSC 1562) and the Oxford Tropical Research Ethics Committee (OXTREC reference 18‐09). The trial was registered at clinicaltrials.gov (NCT00934492). Analysis of the KCH admissions and KHDSS data was approved by the Kenya Medical Research Institute (KEMRI) Scientific Ethics Review Unit (SCC 2778).

## 3 RESULTS

### 3.1 Study participants

In the original randomised trial, 1,778 children with cSAM were enrolled when they had reached stabilisation. Sixty children (3.4%) died, and two withdrew consent before index discharge and were excluded from this analysis. Of the 1,716 children discharged alive and followed up (Figure 1a), nine children were lost to follow‐up, and three withdrew consent within 2 weeks after discharge and were also excluded from this analysis. We therefore included 1,704 children in follow‐up for a total of 1,504.19 child‐years of observation (Figure 1a). Their median age (interquartile ratio) was 11 (7–16) months, and 812 (49%) were female. The mean (SD) MUAC was 10.6 (1.06) cm, and 285 (17%) had kwashiorkor. Baseline study participant characteristics are shown in Table 1.

**Table 1:** Characteristics of participants in the randomised trial analysed in this study

Characteristics	
*n*	1,704
Median age (mo.) at admission [IQR]	11 [7–16]
Girls *n* (%)	812 (49)
Nutritional oedema *n* (%)	285 (17)
MUAC^a^, cm mean ± SD	10.6 ± 1.1
Weight‐for‐length *z*‐score ± SD^b^	−3.3 ± 1.2
Length‐for‐age *z*‐score ± SD	−2.9 ± 1.7
Recruitment site *n* (%)	
Kilifi	145 (8)
Malindi	255 (15)
Mbagathi	489 (29)
Mombasa	812 (48)
*n* randomised to co‐trimoxazole prophylaxis	851 (50)
Post‐discharge mortality *n* (%)^c^	197 (12)

Abbreviation: IQR, interquartile ratio; mo., month; SD, standard deviation.

a Mid‐upper arm circumference.

b Excluding kwashiorkor cases.

c Children were recruited after “stabilisation” phase hence the low inpatient mortality.

A total of 31,807 children were admitted to the KCH from 2002 to 2016, of which 7,168 (23%) met the inclusion criteria for cSAM; 5,515 (77%) were marasmic; and 1653 (23%) had kwashiorkor. Of the children admitted with cSAM, 945 (13%) died before discharge and were excluded. Furthermore, 3,595 children who lived outside the KHDSS surveillance area and nine patients who had missing anthropometry at index admission were also excluded from the analysis. We, therefore, included 2,651 children in this analysis (Figure 1b). Their median age (interquartile ratio) was 13 (6–23) months, and 1,199 (45%) were female. The mean MUAC (including kwashiorkor cases) was 11.5 cm (SD 1.8), and 509 (19%) had oedema (supporting information Table S1).

### 3.2 Association between SAM phenotype at index admission and phenotype upon readmission with cSAM

**Figure 2:**
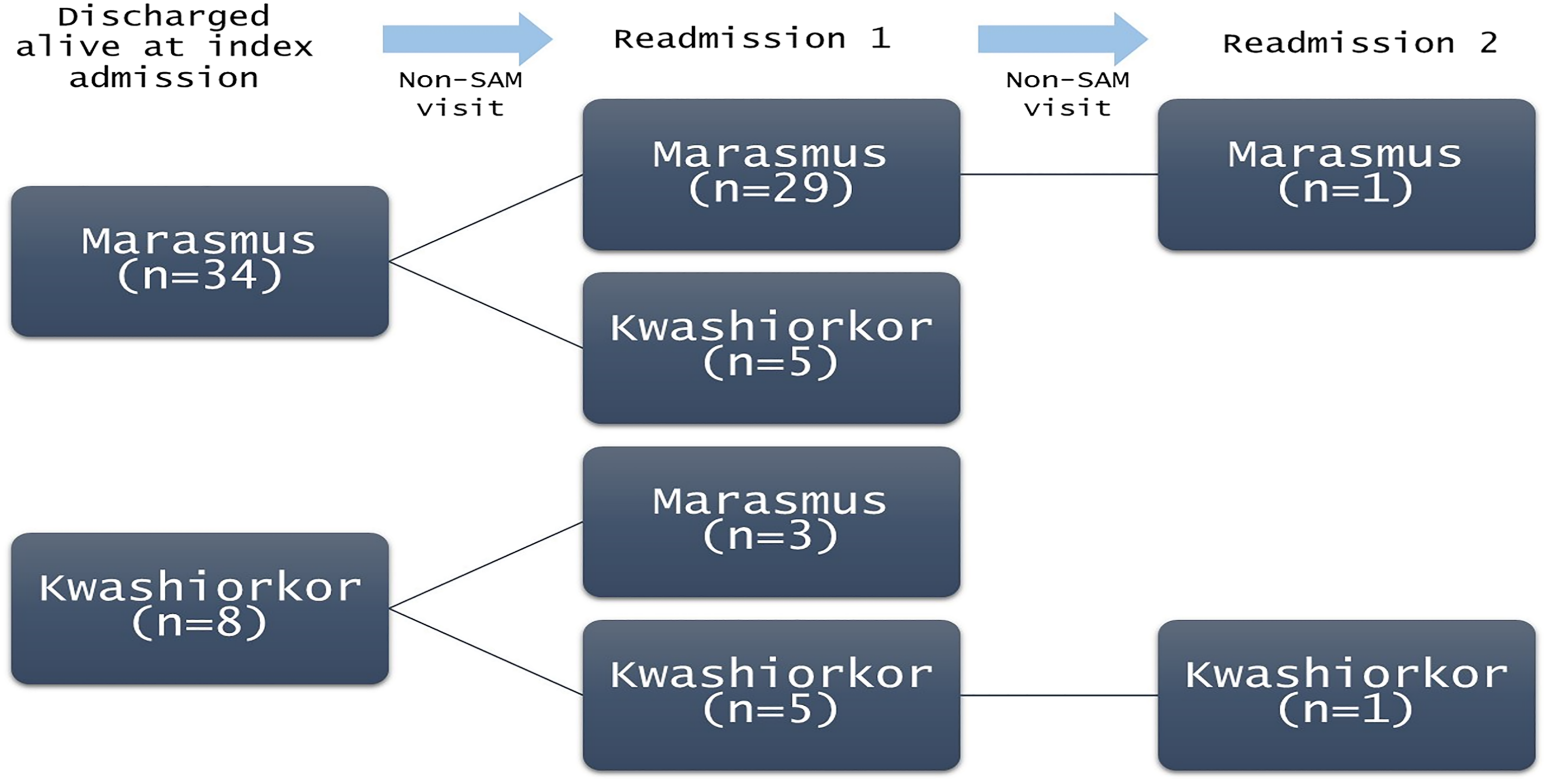
Phenotype (kwashiorkor or marasmus) during index admission and during readmissions after nutritional recovery. A total of 42 children were readmitted with severe acute malnutrition (SAM) after nutritional recovery, and 2 children were readmitted with SAM twice. The second readmission with SAM occurred after 1‐month follow‐up where the child was not SAM

There were 246 readmissions with cSAM in the trial. Of these, 42 children were readmitted with cSAM readmission 44 times after a monthly visit where they were confirmed not to have SAM prior to being readmitted; 34 and 8 had marasmus and kwashiorkor at index admission, respectively. Of these children, 29/34 (85%) and 5/8 (62%) admitted with marasmus and kwashiorkor, respectively, at index presented with the same phenotype upon readmission with cSAM (Figure 2). The adjusted odds ratio of presenting with kwashiorkor during readmission when the patient had kwashiorkor at index admission was 39.3 [95% confidence interval (95% CI) [2.69, 1326]; *p* = .01]. On the other hand, the adjusted odds ratio of presenting with marasmus during readmission when having kwashiorkor at index admission was odds ratio 0.02 (95% CI [0.001, 0.32]), *p* = .01. Phenotypic concordance was not influenced by age (*p* = .19), sex (*p* = .14), season (*p* = .98), nor arm in the clinical trial (*p* = .93). Recruitment site was set as a random effect in the mixed logistic model.

**Figure 3:**
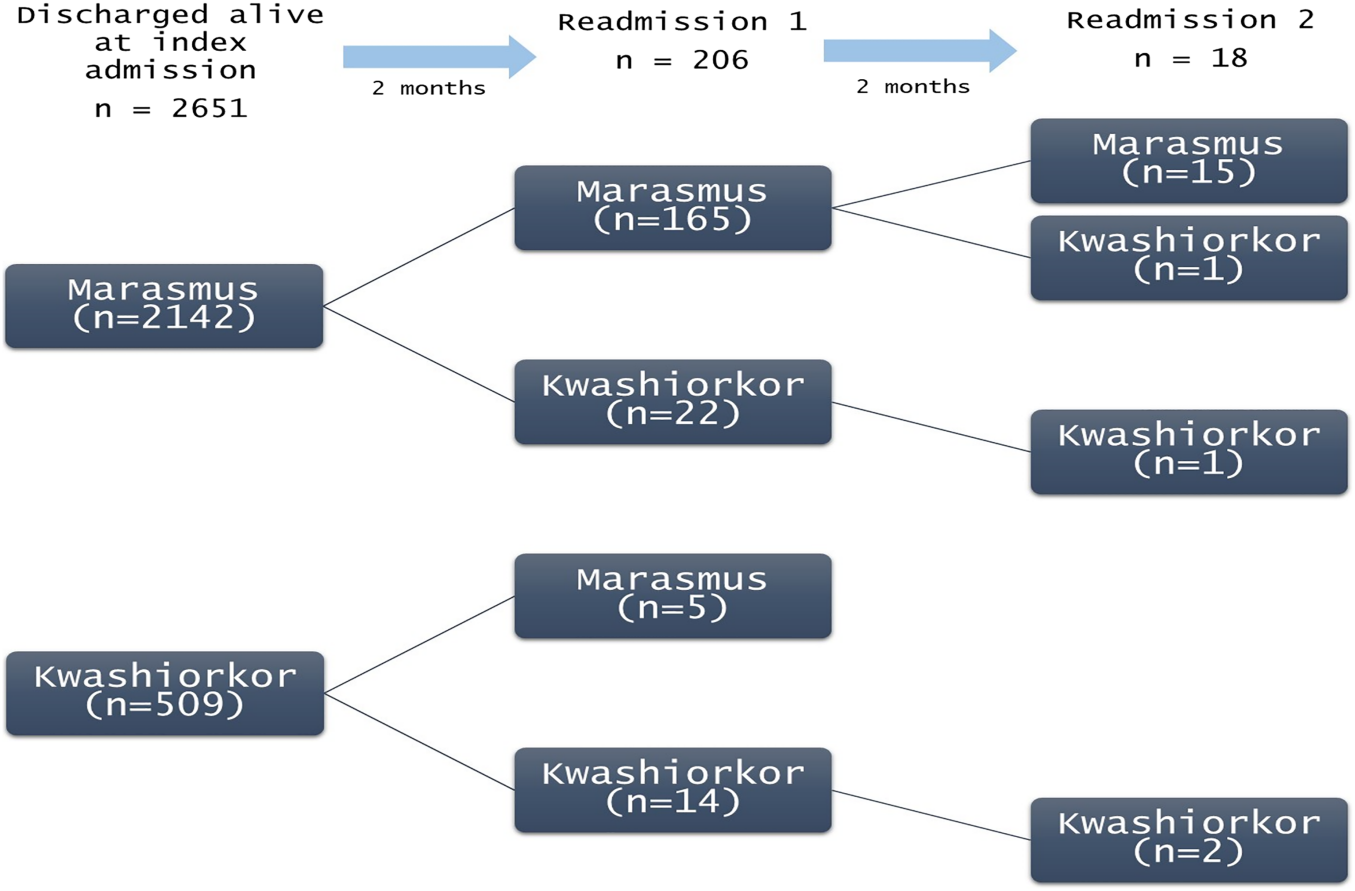
Phenotype (kwashiorkor or marasmus) during index admission and during consecutive complicated severe acute malnutrition readmissions with at least 2 months between admissions based on admissions data of the Kilifi County Hospital from 2002–2016 of children admitted with severe acute malnutrition aged 2–59 months residing within the scope of the Kilifi Health Demographic Surveillance and Survey

Examining the KCH admissions data for validation, we included 2,651 children in the analysis that comprised those discharged from KCH after cSAM treatment and residing within the KHDSS area. A total of 206 (7.8%) children were readmitted with SAM between 2 and 12 months postdischarge. Of the 2,412 children discharged alive with marasmus at index, 187 (7.8%) were readmitted with cSAM. A majority (165/187, 88%) retained marasmus status at the readmission whereas 22/187 (12%) returned with kwashiorkor on their first readmission (Figure 3). Of the 509 children discharged alive with kwashiorkor at index admission, 19 (3.7%) were readmitted with cSAM. A majority (14/19, 74%) were readmitted with kwashiorkor and 5/19 (23%) returned with marasmus. Consistent with the results obtained from the randomised trial data, there was a significant association (*p* < .001) between phenotype at index admission and the phenotype upon readmission with cSAM, accounting for age, sex, and MUAC at index admission. This association was also observed between first readmission and second readmission (*p* = .03) separated by at least 2 months.

### 3.3 Readmissions with complicated severe acute malnutrition

**Figure 4:**
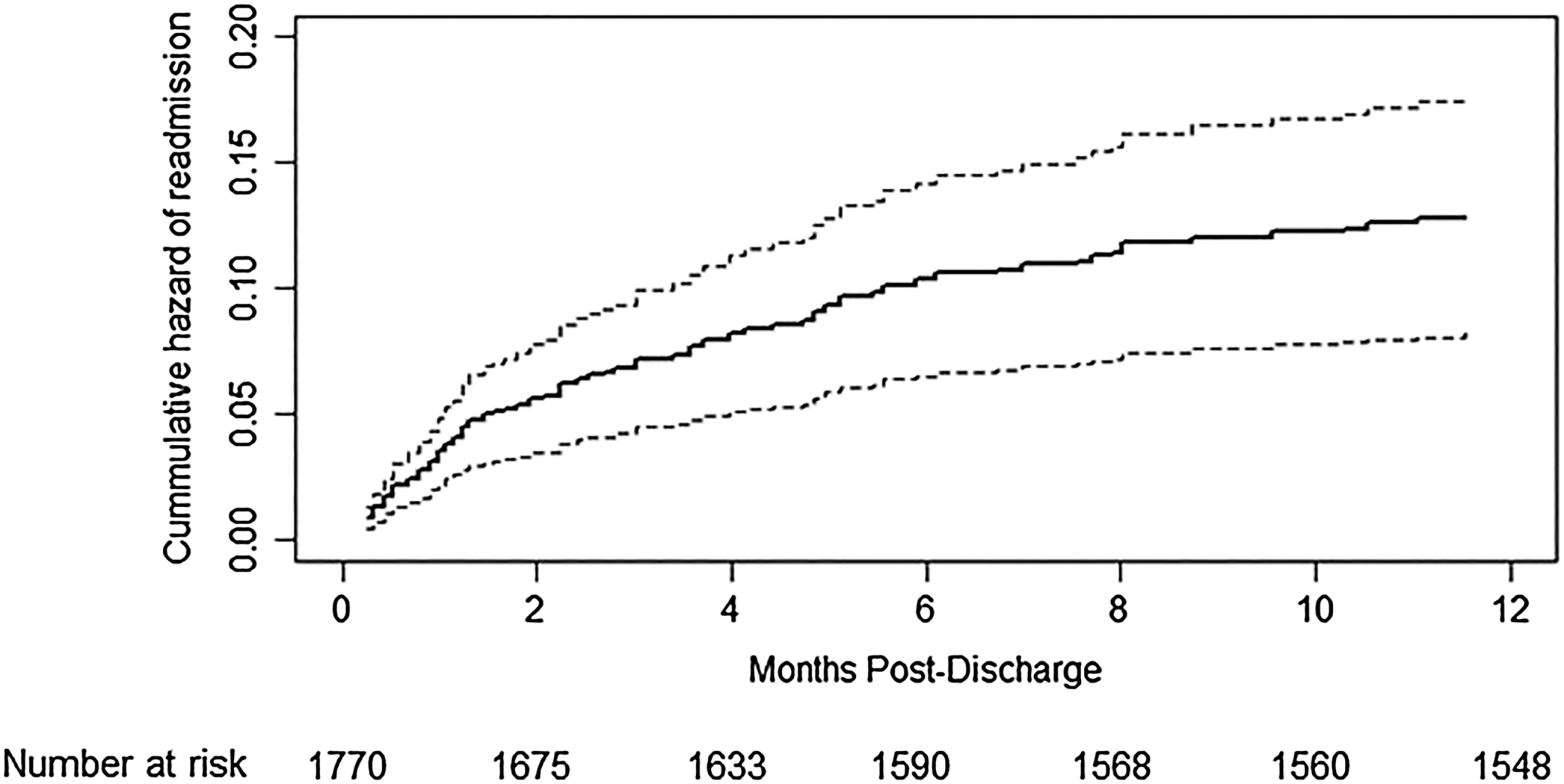
Cumulative hazard of readmission with cSAM based on the Fine‐Gray model. Broken lines signify 95% confidence intervals

In the trial, a total of 729 readmissions occurred among 658/1704 (37%) children during follow‐up. Of the 729 readmissions, 246 readmissions involved cSAM among 174/1704 (10.2%) children during 1,504.19 child years of follow‐up, a rate of 15.9 (95% CI [13.9, 18.0]) per 100 child years. One‐hundred and thirteen (113) children were readmitted once with cSAM, 57 twice, and 3 children were readmitted with cSAM thrice and one 4 times. We found no significant association between SAM phenotype at index admission and rate of cSAM readmission (adjusted subdistribution hazards ratios = 1.3, 95% CI [0.8, 2.3] *p* = .32) after adjusting for age, sex, site, season and arm in the randomised trial (Table 2). Age however, especially being less than 12 months old, was significantly associated with cSAM readmission. Figure 4 shows the cumulative hazard plot for cSAM readmission, which shows the risk of any readmission with cSAM is 12%, within 1 year postdischarge.

**Table 2:** Estimates for the Fine‐Gray hazard model to assess rates of hospital readmissions with complicated severe acute malnutrition accounting for competing risks in the clinical trial (death and lost‐to‐follow‐up)

Covariates	Sub‐distribution hazard ratio	95% Confidence interval	Robust standard error	*p*
Marasmus	Reference			
Kwashiorkor	1.31	0.76–2.26	1.31	.32
Age:				
<1 year	1.73	1.13–2.64	1.24	<.01
1–2 years	Reference			
>2 years	1.40	0.75–2.63	1.37	.28
Female	Reference			
Male	1.07	0.74–1.52	1.19	.42
Site:				
Kilifi	Reference			
Malindi	0.62	0.29–1.31	1.46	.21
Mbagathi	0.97	0.49–1.90	1.40	.93
Mombasa	0.80	0.42–1.50	1.38	.49
Season:				
Dry	Reference			
Rainy	1.31	0.93–1.83	1.19	.11
Arm in original trial				
Treatment	Reference			
Placebo	1.29	0.92–1.81	1.89	.14

## 4 DISCUSSION

This study aimed to test the association between cSAM phenotype at index admission and readmission following recovery. Our results showed an association between the phenotype at index admission and readmission phenotype for cSAM indicating that SAM phenotype persists in the same children during relapses. Findings on phenotype persistence were confirmed in a separate validation data set comprising of children admitted to a rural county hospital in a natural setting. It is possible that SAM phenotype could be persistent due to the fact that the child goes back to the same environment, household conditions, and perhaps dietary habits after discharge from the hospital. However, a genetic predisposition to a certain phenotype has previously been suggested, relating to disruption of intestinal sulphated glycosaminoglycans in kwashiorkor (and not in marasmus), which the authors ascribed to a potentially genetically influenced lower sulphated glycosaminoglycans synthesis rate (Amadi et al., 2009). The role of the gut microbial composition also cannot be discounted as children with SAM have been found to harbour a persistent immature gut microbiota composition even after nutritional intervention (Subramanian et al., 2014), which could also influence the phenotype that a child with SAM relapses. We also found that the index admission phenotype of marasmus or kwashiorkor did not alter the risk of readmission with cSAM, but children younger than 1 year old were more likely to be readmitted with cSAM.

Of the few studies that examined nutritional and clinical rehabilitation of children with SAM treated in nutritional rehabilitation units or hospital facilities, reported incidences where children relapsed to SAM varied from 0–13% (Beau, 1993; Briend et al., 2005; Khanum, Ashworth, & Huttly, 1998; Pécoul, Soutif, Hounkpevi, & Ducos, 1992; Perra & Costello, 1995; van Roosmalen‐Wiebenga, Kusin, & de With, 1987). However, in these studies, it is unclear whether these children also developed clinical complications in addition to SAM because they were conducted prior to the current classification of complicated and uncomplicated SAM. Two studies explicitly noted that children that relapsed to SAM were readmitted to the hospital although any clinical comorbidities were not specified (Khanum et al., 1998; van Roosmalen‐Wiebenga et al., 1987). In the study of Khanum et al. (1998), children that developed oedema or with <60% of weight‐for‐height were readmitted together with nonmalnourished children with medical emergencies, although it was unclear whether the malnourished children needed hospital care. In the study of van Roosmalen‐Wiebenga et al. (1987), children that relapsed were readmitted to the hospital, although definition of relapse and their discharge criteria were unclear. Treatment guidelines have also changed since 1987, which means that the results of their study may need to be re‐evaluated. Although data on the mortality and relapse to SAM of children managed in Community‐Based Management of Acute Malnutrition programmes (uncomplicated SAM) have been reported (O'Sullivan, Lelijveld, Rutishauser‐Perera, Kerac, & James, 2018; Stobaugh et al., 2018), only a few papers reported nutritional outcomes of children after treatment from cSAM (Fergusson et al., 2009; Kerac et al., 2009; Kerac et al., 2014; Lelijveld et al., 2016).

The goal of SAM treatment also includes the reduction of susceptibility to life‐threatening infections, restoration of a healthy body composition, and improvement in neurocognitive status (Ngari et al., 2018a). However, children with SAM remain at risk of developing severe illness and/or infections after treatment of clinical complications and discharged from hospital (Berkley et al., 2016; Chisti et al., 2014; Kerac et al., 2014; Ngari et al., 2018a). A study by Kerac et al. (2014) that followed 1,024 children 1 year post‐discharge reported 5% hospital readmissions but 42% deaths, whereas Khanum et al. (1998) reported 1.2% emergency hospital readmissions. These studies however only captured one readmission event per child. Accounting for multiple hospital readmissions, a previous report recorded 616 nonfatal admissions to hospital and 3,266 nonfatal episodes of illness post‐discharge among 1,778 children in the original randomised trial (Berkley et al., 2016). A secondary analysis of this data focusing on life‐threatening events reported 823 life‐threatening events (257 fatal and 566 nonfatal events) among 612 children after discharge from the hospital (Ngari et al., 2018a), including a high rate of events in those who had recovered their nutritional status. However, these reports did not focus on the children who specifically readmitted with cSAM. In this current study, we found that that by 1 year discharge, the risk of any readmission with cSAM is 12%, which we believe is within the range that would be reasonably anticipated in a poor, food‐insecure population.

This is the first report that assessed the association between SAM phenotype at index admission with the phenotype at succeeding readmissions. The randomised control trial had a very low dropout rate (5%); as such, we were able to capture multiple readmissions of children even for those who were readmitted to different hospitals. However, because the randomised trial did not include children with HIV, we cannot generalise the study results on readmission rates to children with SAM and HIV, a common feature in Africa, with a known elevated mortality and deterioration risk (Kerac et al., 2014). Furthermore, we were unable to assess compliance to outpatient therapeutic programme.

Systematic HIV screening at the KCH was implemented in 2007, as such, some of the children included in the KCH data analysis were known to have HIV. However, since we only performed passive follow‐up on these children, readmission rates, including those to other hospitals, could not be reliably not estimated. Furthermore, because these children were not actively followed, we only considered a readmission when it occurred at least 2 months after the previous hospital discharge.

## 5 CONCLUSION

This is the first report of hospital readmissions with cSAM showing that SAM phenotype at index admission is sustained at succeeding readmissions with cSAM.

## CONFLICTS OF INTEREST

The authors declare that they have no conflicts of interest.

## CONTRIBUTIONS

GBG designed the study, performed statistical analysis and writing of the first manuscript draft. MMN and JMN provided advise on design, analysis and interpretation of results and contributed to the writing of the revised drafts. JT, LM, NM, MM, CO and AN were responsible for clinical care and data collection. JAB was the principal investigator of the parent trial and offered overall supervision in design, analysis, and interpretation of the study results. All authors reviewed and agreed on the final manuscript.

## Supporting information

Table S1. Characteristics of participants in the Kilifi County Hospital database included in this study
