# Corrigendum

**DOI:** 10.1111/mcn.13007

**Published:** 2020-06-16

In the following article (Gonzales et al., 2020), there was a typesetting error in key messages on page 2. The word “readmitted” should have been changed to “admitted” in the sentence.

The incorrect sentence reads:

Severe malnutrition phenotype was persistent such that a child readmitted for kwashiorkor would be likely to be readmitted with kwashiorkor again.

The correct sentence should have read:

Severe malnutrition phenotype was persistent such that a child admitted for kwashiorkor would be likely to be readmitted with kwashiorkor again.
