# Supplementary material for: Phenotype is sustained during hospital readmissions following treatment for complicated severe malnutrition among Kenyan children: A retrospective cohort study
Source: Matern Child Nutr. 2019 Nov 22;16(2):e12913. doi: 10.1111/mcn.12913 (PMC7083470; doi:10.1111/mcn.12913)
Supplement: Supplementary file 1 — Table S1. Characteristics of participants in the Kilifi County Hospital database included in this study [file MCN-16-e12913-s001.docx]

Supplementary table 1. **Characteristics of participants in the Kilifi County Hospital database included in this study**

|  | **KCH KHDSS-resident dataset** |
| --- | --- |
| *N* | 2651 |
| Median age (mo.) at admission [IQR] | 13 [6 – 23] |
| Girls *n* (%) | 1199 (45) |
| Nutritional oedema *n* (%) | 509 (19) |
| MUAC^$^, cm mean ± SD | 11.2 ± 1.9 |
| Weight-for-height z-score* ± SD | -3.1 ± 1.4 |
| Length-for-age z-score ± SD | -2.4 ± 2.0 |
| HIV seropositive (since 2007) *n* (%)^§^ | 110/1427 (7) |

^$^Mid-upper arm circumference, *Excluding kwashiorkor cases, ^§^Systematic HIV screening program started in 2007
